# Novel “on–off” fluorescence sensing for rapid and accurate determination of Cr^3+^ based on g-CNQDs†

**DOI:** 10.1039/d3ra05091b

**Published:** 2023-09-28

**Authors:** Xiaohua Xu, Huye Li, Yapeng Sun, Tianfeng Ma, Lin Shi, Wencheng Mu, Huan Wang, Yongchang Lu

**Affiliations:** a Modern Tibetan Medicine Creation Engineering Technology Research Center of Qinghai Province, College of Pharmacy, Qinghai Nationalities University Xining 810007 China qhmuwh1028@126.com qhlych@126.com; b The Fourth People's Hospital of Qinghai Province Xining 810007 China; c No. 2 Middle School in Xining City Xining 810007 Qinghai Province China

## Abstract

Cr^3+^ is one of the most essential trace elements in living organisms and plays a vital role in human metabolism. However, both deficiency and excess intake of Cr^3+^ can be harmful to the human body. Therefore, the quantitative determination of Cr^3+^ is of great significance in the field of life science. Based on this, in this study, a g-CNQDs@*p*-acetaminophenol fluorescence sensing system was developed for the quantitative detection of Cr^3+^ in actual complex samples. G-CNQDs were synthesized with sodium citrate and urea as precursors. The fluorescence signal was enhanced by the synergistic effect between *p*-acetaminophenol (APAP) and g-CNQDs. The fluorescence quenching phenomenon can be produced when Cr^3+^ is introduced into the fluorescence-enhanced g-CNQDs@*p*-acetaminophenol system. An “on–off” fluorescence sensing system was constructed based on g-CNQDs@*p*-acetaminophenol for the quantitative detection of Cr^3+^. The experimental data showed a wide linear region in the concentration range of 0.64–63.0 μM, and the detection limit was as low as 0.23 μM. The construction of the sensor system broadens the research field for the practical application of Cr^3+^.

## Abstract 2

An “on–off” fluorescence sensing system was constructed with g-CNQDs@*p*-acetaminophenol for detection of Cr^3+^. g-CNQDs were synthesized, and the fluorescence signal was enhanced based on the effect between APAP and g-CNQDs. The introduction of Cr^3+^ into the system results in fluorescence quenching.

## Graphical abstract

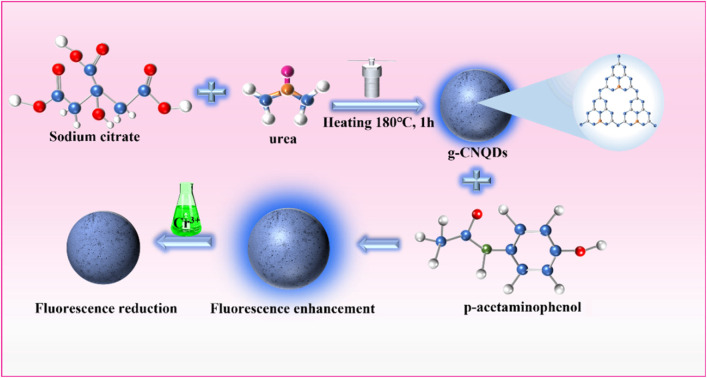

## 1 Introduction

Cr^3+^ is one of the essential trace elements in humans and animals and has a direct impact on the metabolism of carbohydrates, fats, proteins, and nucleic acids.^1,2^ The lack of Cr^3+^ has been reported to cause vascular disease, diabetes, *etc.*, while excessive intake can damage cell composition and cause harm to the body, causing disorders of blood glucose and lipid metabolism.^3–6^ It is well known that the human body needs a small amount of Cr^3+^, which can only be taken from food due to its inability to synthesize it. To prevent the lack and excessive intake of Cr^3+^, the Chinese Nutrition Society recommends a daily dietary Cr^3+^ intake of 50–200 μg for adults,^7^ and the United States Environmental Protection Agency standard limit for Cr^3+^ in drinking water is 0.1 mg mL^−1^.^6^ In addition, Cr^3+^ is easily oxidized to Cr^6+^ by oxidants. Cr^6+^ has higher toxicity compared with Cr^3+^, which can cause allergy through skin contact, and easy to cause genetic defects or cancer after inhalation. It can cause long-lasting harm to the environment when Cr^6+^ is discharged into the environment.^8–10^ Therefore, it is urgently required to construct a simple, efficient, and selective method for the quantitative detection of Cr^3+^ in the field of environmental and biological health monitoring.

At present, various detection techniques have been successfully developed for the determination of Cr^3+^, including chromatography,^11,12^ spectrophotometry,^13,14^ inductively coupled plasma-mass spectrometry (ICP-MS),^15–17^ atomic absorption spectrometry,^18,19^ and electrochemical methods.^20,21^ Although these methods have high accuracy and sensitivity, they still have disadvantages, such as expensive equipment, complicated sample pretreatment, and time-consuming, which limit their wide application to some extent. Therefore, finding a simple, fast, and sensitive analytical technique for Cr^3+^ has become a hot research topic. In contrast, fluorescence spectrophotometry represented by carbon quantum dots is widely popular because of its low cost, simple operation, fast response, remarkable selectivity, and high sensitivity.^6,22^ Therefore, it is of great significance to construct novel fluorescent sensors to detect Cr^3+^ for accurate and selective identification and quantitative monitoring of Cr^3+^. In addition, it has been reported that fluorescence sensing systems can be used to detect Cr^3+^ in a low-cost, ultra-sensitive, simple, and rapid way. Krishnan *et al.* designed and synthesized a new diphenylimidazole-based fluorescent probe 5, which realizes the dual sensing of Hg^2+^ and Cr^3+^ ions. It also has been successfully used for the detection of Cr^3+^ in environmental water samples and *E. coli* bacteria cells.^6^ Wang *et al.* designed a chemiluminescent sensor L based on a coumarin base, which could realize the rapid and selective detection of Cr^3+^. The cell imaging and real-time monitoring of Cr^3+^ in living HepG2 cells have been successfully realized due to its good water solubility and biocompatibility.^23^

In recent years, semiconductor quantum dot (SQD) materials have gained wide applications in bioimaging and biosensors owing to their unique optical properties.^24^ However, some common quantum dots, such as cadmium sulfide (CdS) and cadmium telluride (CdTe), have certain toxicities that will have harmful effects on human health and the environment.^25^ Compared with traditional organic dyes and semiconductor quantum dots, graphitic carbon nitride quantum dots (g-CNQDs), as an emerging non-metallic semiconductor nanomaterial, not only maintain good water solubility, chemical stability, biocompatibility, and high photoluminescence quantum yield but also have the advantages of low cost, easy synthesis, non-toxicity, and green environmental protection. g-CNQDs have shown great promise for applications in photocatalysis, chemical and biological sensing, bioimaging, and drug delivery owing to their excellent optical properties.^26,27^ These excellent properties make it possible to replace conventional quantum dots in biological detection. To date, a number of fluorescent sensors based on g-CNQDs have been developed for the detection of biological ions. For example, Wang *et al.* used a simple one-step hydrothermal method to synthesize blue fluorescent carbon quantum dots for the highly sensitive detection of the environmental pollutant Cr^6+^.^28^ Guo *et al.* prepared a novel nanoprobe based on S, O-doped carbon nitride quantum dots (S,O-CNQDs), which show great potential for the detection of folate and targeted imaging of cancer cells.^29^ In conclusion, g-CNQDs can be used as a fast, simple, and highly selective fluorescent sensing probe for the detection of biological and environmental samples because of their good physicochemical properties.

**Fig. 1:**
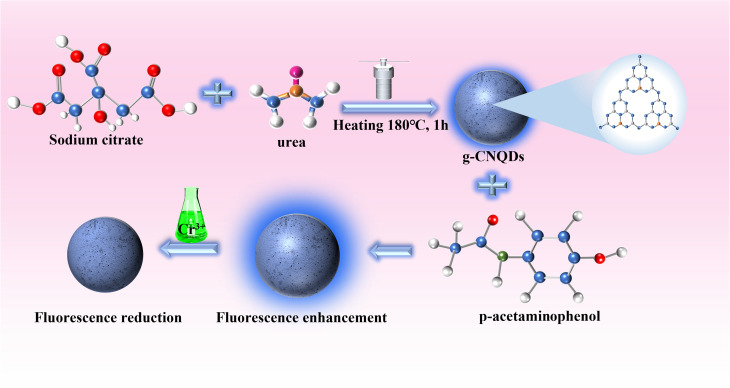
Research scheme of Cr^3+^ sensing of g-CNQDs@*p*-acetaminophenol system.

In this study, g-CNQDs were synthesized and used for Cr^3+^ detection (Fig. 1). g-CNQDs were prepared using the low-temperature solid phase reaction method.^30^ The synthesis method has the characteristics of a simple process, low energy consumption, and low pollution. The maximum fluorescence emission spectrum of g-CNQDs at 446 nm was enhanced due to the synergistic effect between g-CNQDs and *p*-acetaminophenol when *p*-acetaminophenol was injected into luminophor. At this time, the sensor system was turned on. The enhanced fluorescence intensity was quenched due to photoinduced electron transfer between g-CNQDs@*p*-acetaminophenol and Cr^3+^ when Cr^3+^ was further introduced into the proposed sensing system. Thus, the sensor study of g-CNQDs@*p*-acetaminophenol on Cr^3+^ was conducted. The quantitative analysis of Cr^3+^ in environmental water samples was successfully performed. Compared with various previously reported detection methods, the fluorescence sensor constructed in this study is simple, sensitive, and efficient. The proposed sensing system exhibits good potential for application in the monitoring of Cr^3+^ in complex environmental samples.

## 2 Experimental

### 2.1 Reagents

All reagents were of analytical reagent grade and were used without further purification. Urea was purchased from the Hedong District of the Tianjin Hongyan Reagent Factory (Tianjin, China). Sodium citrate was purchased from the Beijing Chemical Factory (Beijing, China). *p*-Acetaminophenol and quinine sulfate were purchased from Aladdin Co., Ltd. (Shanghai, China). Sodium dihydrogen phosphate, disodium hydrogen phosphate, sodium chloride, H_2_O_2_, and chromium chloride (CrCl_3_·6H_2_O) were purchased from the Tianjin Damao Chemical Reagent Factory (Tianjin, China). The preparation reagents for the metal ion solutions were purchased from Sinopharm Chemical Reagent Co., Ltd. (Shenyang, China). Anhydrous ethanol was purchased from Xinxiang Zhengxin Chemical Co., Ltd. (Henan, China).

### 2.2 Characterization methods

Transmission electron microscopy (TEM) and high-resolution TEM (HRTEM) images of g-CNQDs were obtained using a JEM-2100 transmission electron microscope (Shimadzu Corporation, Japan) at an accelerating voltage of 200 kV. The X-ray diffraction (XRD) analysis of g-CNQDs was performed using an X-ray powder diffractometer (PANalytical B.V., Netherlands). The elemental composition and proportion of g-CNQDs were obtained using X-ray photoelectron spectroscopy (XPS) using a D8AA25 X-ray photoelectron spectrometer (Bruker Corporation, Germany). The fluorescence properties of g-CNQDs were determined using an RF-5301PC fluorescence spectrophotometer (Shimadzu Corporation, Japan). UV-Vis absorption spectroscopy was performed using a TU-1901 double-beam UV-Vis spectrophotometer (Beijing Purkinje General Instrument Co., Ltd., China). Fourier transform infrared spectroscopy (FT-IR) was carried out using a Nicolet IS-10 FT-IR spectrometer (Thermo Fisher, USA). The pH of the PBS buffer solution was determined using a pHS-2C digital pH meter (Ridao Science Instrument Co., Ltd., China).

### 2.3 Preparation of materials

#### 2.3.1 Synthesis of g-CNQDs

g-CNQDs were prepared using an improved low-temperature solid-phase reaction method using trisodium citrate as the carbon source and urea as the nitrogen source.^30^ Briefly, 1.68 mmol urea and 0.28 mmol trisodium citrate were mixed and pressed into a fine powder in a 6 cm agate mortar. The mixture was moved into a stainless steel autoclave containing a polytetrafluoroethylene liner and then heated to 180 °C in a blast-drying oven for 2 h. Then, the reaction was reduced to room temperature to obtain a yellow mixture. After the mixture was washed and purified with anhydrous ethanol for five times, pale yellow g-CNQDs nanomaterials were obtained. Subsequently, the obtained g-CNQDs were dialyzed in water for 24 h to remove some interferences (*e.g.*, C_5_H_5_O_5_COO^−^, Na^+^). The obtained products were dried in an oven at 60 °C, and a yellow solid powder of pure g-CNQDs was obtained. Finally, the yellow solid powder was stored in a refrigerator at 4 °C for further characterization and application.

#### 2.3.2 Preparation of the phosphate buffered solution

1.3609 g potassium dihydrogen phosphate and 2.2822 g dipotassium hydrogen phosphate were dissolved in ultrapure water, and transferred in a 100 mL volumetric flask to obtain 0.1 mol L^−1^ buffer solution; different pH values were obtained by mixing different proportions of potassium dihydrogen phosphate and dipotassium hydrogen phosphate.

### 2.4 Interference experiment with the Cr^3+^ fluorescent sensor

In order to explore the sensing performance of g-CNQDs, 13 metal ions (Al^3+^, K^+^, Mg^2+^, Ni^2+^, Na^+^, Hg^2+^, Mn^2+^, Pb^2+^, Cu^2+^, Zn^2+^, Ba^2+^, Cd^2+^, and Cr^3+^) with concentrations of 1 mmol L^−1^ were prepared to evaluate the selectivity of the fluorescent sensor. The optimized g-CNQDs and *p*-acetaminophenol solutions were taken and added to a quartz cuvette to make a mixed solution. Then, the above metal ions were added to the mixed solution separately. After incubation for 1 min at room temperature, the fluorescence spectra were monitored at the excitation wavelength of 340 nm to evaluate if the presence of other ions interfered with the quenching effect of Cr^3+^.

### 2.5 Detection of Cr^3+^ in the actual samples

The environmental water samples used in the experiments were obtained from the laboratory of Qinghai University for Nationalities. The serum in the actual sample analysis was provided by the Fourth People's Hospital of Qinghai Province. Before analysis, all samples were centrifuged at 8000 rpm for 5 min and then filtered using a 0.22 μm filter membrane. The prepared 3 mL g-CNQDs@*p*-acetaminophenol solution was added to the above water sample solution at different volumes. The fluorescence intensity was detected after the reaction at room temperature for 3 min. The reliability and accuracy of g-CNQDs@*p*-acetaminophenol fluorescence sensor for the detection of Cr^3+^ were verified by standard recovery experiments.

## 3 Results and discussion

### 3.1 Characterization of the prepared g-CNQDs

**Fig. 2:**
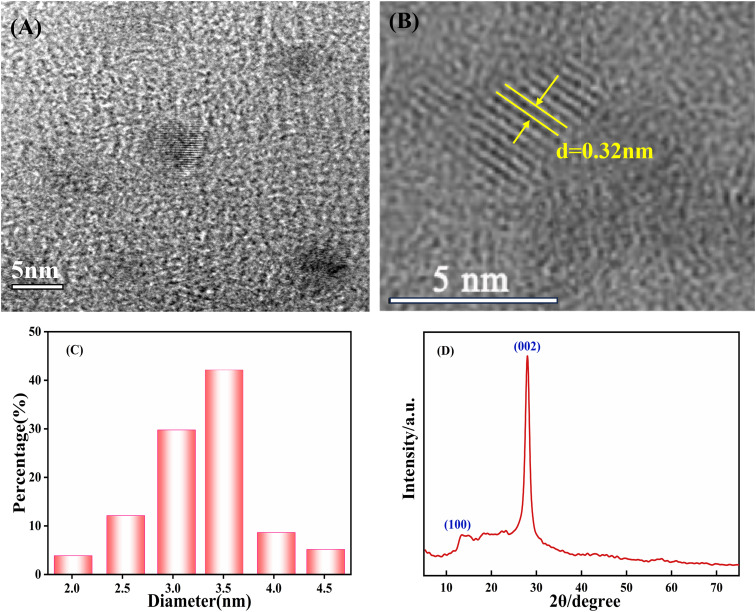
(A) TEM images, (B) HR-TEM images, (C) particle size distribution histogram, and (D) XRD of the prepared g-CNQDs.

g-CNQDs were synthesized by a simple low-temperature solid-phase reaction using trisodium citrate and urea as raw materials. To prove the successful synthesis of g-CNQDs, the prepared g-CNQDs were characterized using TEM, XRD, FT-IR, and XPS. TEM was used to characterize the morphology, particle size, and dispersion of the synthesized g-CNQDs, as shown in Fig. 2. Through TEM characterization at 5 nm (Fig. 2A) and HR-TEM images (Fig. 2B), it was shown that the prepared g-CNQDs had a spherical morphology, uniform particle size, and good dispersion. From HR-TEM (Fig. 2B), it can be clearly observed that the prepared g-CNQDs have obvious crystal characteristics, and the lattice spacing was measured at 0.32 nm. Fig. 2C shows that 108 nanoparticles were selected and added to the statistics using the ImageJ software to determine the particle size of g-CNQDs. It can be seen that the particle size distribution of g-CNQDs ranges from 2 nm to 4.5 nm with an average particle size of 3.3 nm. The XRD characterization shown in Fig. 2D further indicated the two characteristic peaks of g-CNQDs, which corresponded to the (100) and (002) crystal planes of pure carbon nitride.^31^

**Fig. 3:**
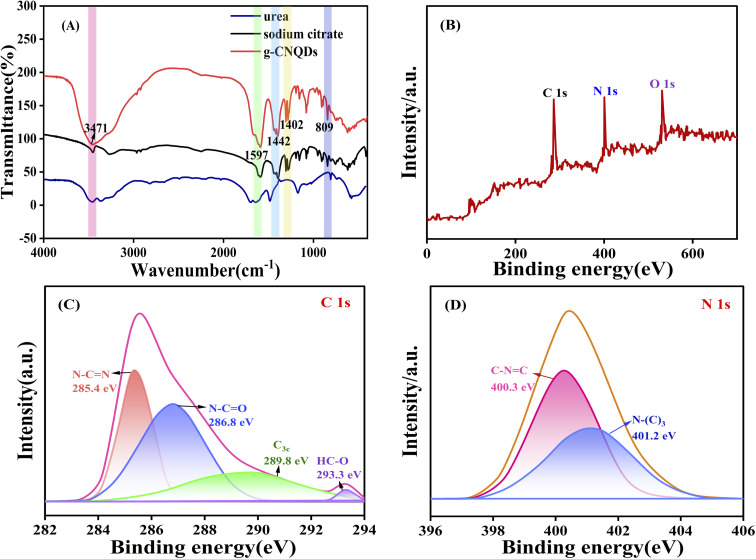
(A) FT-IR spectra of g-CNQDs (g-CNQDs is the red line, sodium citrate is the black line, and urea is the blue line), XPS full spectra (B), and high-resolution XPS spectra of C 1s (C) and N 1s (D) of the prepared g-CNQDs.

The surface functional groups of the prepared g-CNQDs were further measured using FT-IR spectroscopy, as shown in Fig. 3A. According to the obtained data, the absorption peak near 811 cm^−1^ was due to the stretching vibration of C–N in the triazine ring.^32^ The absorption bands at 1402 and 1442 cm^−1^ were attributed to the typical stretching vibrations of C=N and C–N bonds on the C–N heterocycles, respectively. The absorption peaks at 3300–3500 cm^−1^ were attributed to the stretching vibrations of N–H and O–H. The high-intensity peak at 1590 cm^−1^ was associated with the C=O asymmetric stretching vibration of the carboxylate anion.^33^ The results indicated that the surfaces of g-CNQDs were modified by amino and hydroxyl groups, which endowed g-CNQDs with good water solubility.

The elemental composition and oxidation states of g-CNQDs were further investigated using XPS. As shown in Fig. 3B, the three XPS peaks at 286.2, 400.5, and 530.8 were attributed to C 1s, N 1s, and O 1s, respectively, indicating the presence of carbon, nitrogen, and oxygen atoms in g-CNQDs. In addition, high-resolution XPS characterization was performed for C 1s and N 1s. The HR-XPS spectra of C 1s in Fig. 3C show three main peaks in 285.4 eV, 286.8 eV, and 289.8 eV. The characteristic peaks at 285.4 eV and 286.8 eV were attributed to N–C=N and N–C=O, respectively. While the peak at 289.8 eV was attributed to C__3c__. A smaller peak at 293.3 eV was attributed to HC–O.^34^ The HR-XPS spectra of N 1s in Fig. 3D show that the N 1s peak was split into two peaks, indicating the presence of two different types of nitrogen in g-CNQDs. The peak at 400.3 eV shows the presence of C–N=C, indicating nitrogen bonded to two carbon atoms. The characteristic peak at 401.2 eV was attributed to quaternary nitrogen bonded by three sp^2^ carbon atoms, called graphitic nitrogen.^35^ The ratio of N_1_/N_2_ was 1 : 40, which was close to the triazine-based graphitized C_3_N_4_. The above results indicate that g-CNQDs based on the C_3_N_4_ structure of the graphite phase were synthesized successfully.

### 3.2 Optical properties of g-CNQDs

**Fig. 4:**
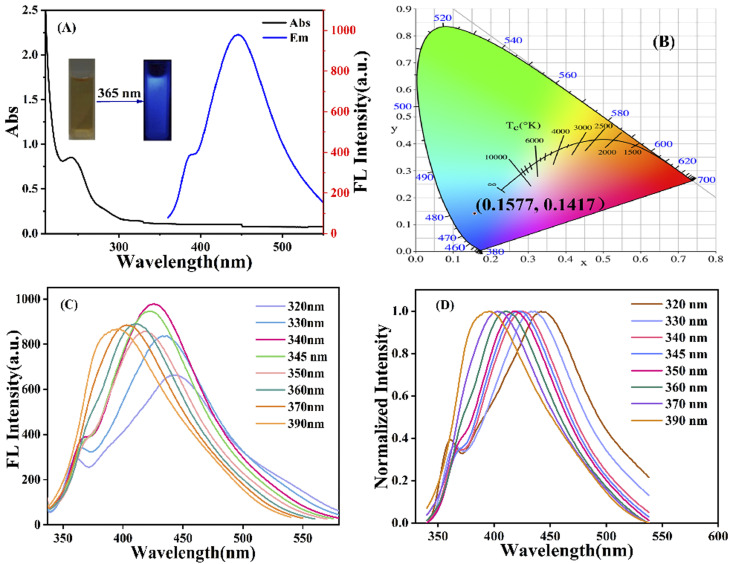
(A) UV-Vis absorption spectra (black line) and emission spectra (blue line) of g-CNQDs, inset: photographs of g-CNQDs solution under visible light (left) and 365 nm UV light (right). (B) CIE colour coordinates of g-CNQDs. (C) Fluorescence emission spectra of g-CNQDs under different excitation wavelengths. (D) Normalized fluorescence spectra of g-CNQDs.

The optical properties of g-CNQDs were investigated using UV-Vis absorption spectroscopy and fluorescence spectroscopy. The UV-Vis absorption spectra of g-CNQDs were studied, as shown in Fig. 4A. The absorption peak at 254 nm in the black line was attributed to the n–π* transition between OH and C–N– on the surface of g-CNQDs.^36^ The fluorescence emission spectrum is represented by the blue line. It is displayed that the maximum fluorescence emission wavelength was obtained at 446 nm when the excitation wavelength of the g-CNQDs solution was 340 nm. The inset illustration indicated that the aqueous solution of g-CNQDs was yellow in visible light (left) and produced blue fluorescence under 365 nm UV light (right), indicating that the fluorescence was derived from the synthesized g-CNQDs. The CIE colour coordinates were simulated (0.1577, 0.1417) using simulation software. The dependence of the excitation wavelength was also examined, as shown in Fig. 4C. The fluorescence intensity initially increased with the increase of the excitation wavelength. The corresponding fluorescence emission intensity showed a decreasing trend when the excitation wavelength reached 340 nm. This meant that the fluorescence emission peak of g-CNQDs had a good excitation wavelength dependence. Fig. 4D shows the normalized fluorescence emission spectra of g-CNQDs at the excitation wavelength of 320–390 nm. The experimental results further proved that the position of the fluorescence emission peak of g-CNQDs gradually redshifted with the increasing excitation wavelength in the range of 320–390 nm.

### 3.3 Study on fluorescence spectral stability of g-CNQDs

In addition, the fluorescence stabilities of g-CNQDs are essential for chemical sensing applications. The effect of NaCl solutions with different concentrations on the fluorescence intensity of g-CNQDs was studied, as shown in Fig. S1A.† Experiments showed that there was no significant change in fluorescence intensity when the concentrations of NaCl solutions were in the range from 0.25 to 1.5 M. At the same time, in order to investigate the antioxidant activity of g-CNQDs, the fluorescence intensity was detected by adding different concentrations of H_2_O_2_ to the g-CNQDs solution, as shown in Fig. S1B.† The results indicated that the fluorescence intensity of g-CNQDs maintained strong stability in the concentration range from 0 to 500 μM H_2_O_2_. In addition, the effects of temperature and UV radiation on the fluorescence intensity of g-CNQDs were also investigated. As can be seen from Fig. S1C,† there was no significant change in the fluorescence intensity of g-CNQDs when the temperature was changed from 25 to 55 °C, indicating that the g-CNQDs had a strong ability to withstand high temperatures. As shown in Fig. S1D,† g-CNQDs still had good photostability even after 90 min of continuous UV irradiation. In conclusion, the prepared g-CNQD fluorescence sensor has excellent resistance to high temperature, salt, oxidation, and photobleaching, which demonstrates that g-CNQDs have good potential for application in the analysis of complex samples.

### 3.4 Construction of sensing system

**Fig. 5:**
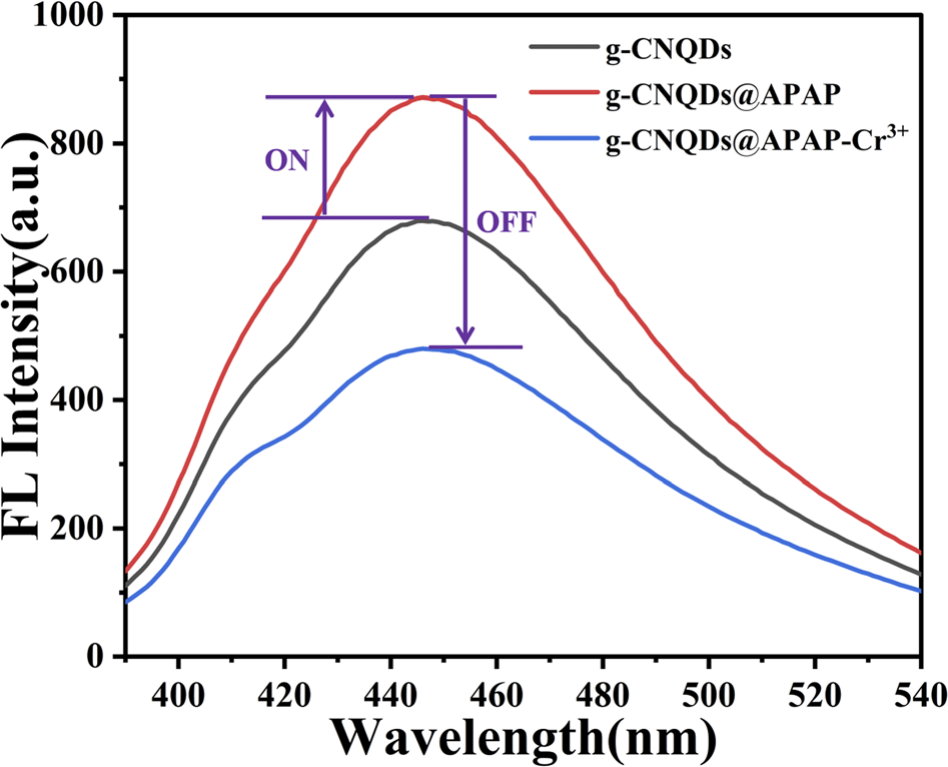
Construction of the “on–off” fluorescence sensing system.

Using g-CNQDs as fluorescent probes, the fluorescence intensity of g-CNQDs was enhanced based on the synergistic effect between *p*-acetaminophenol and g-CNQDs. The enhanced fluorescence intensity was further quenched based on the specific reaction between the *p*-acetaminophenol and Cr^3+^ when Cr^3+^ was further introduced into g-CNQDs@*p*-acetaminophenol (Fig. 5). Therefore, a fluorescence sensing system for Cr^3+^ was constructed based on g-CNQDs@*p*-acetaminophenol.

### 3.5 Optimization of analysis parameters

In order to explore the chemical stability of g-CNQD aqueous solution and the sensitivity of the detection method, the effects of the pH of the buffer solution, concentration of *p*-acetaminophenol, reaction time between g-CNQDs and *p*-acetaminophenol, and reaction time between Cr^3+^ and *p*-acetaminophenol on the fluorescence intensity of g-CNQDs were investigated. The optimization of the experimental conditions was carried out to select the best parameters for the detection of complex samples.

Different pH values of the phosphate buffer solution may have a significant effect on the fluorescence intensity of the fluorescence sensing system. Considering that the buffer solution affects the fluorescence intensity of g-CNQDs, different pH values of PBS were optimized in the fluorescence sensing system, as shown in Fig. S2.† The fluorescence intensity increased with pH increasing from 6 to 7.5. g-CNQDs exhibited the strongest fluorescence intensity when pH was 7.5. The fluorescence intensity gradually decreased with further increase in pH. Therefore, PBS with pH = 7.5 was used for the aqueous medium for further experimental studies.

In addition, as an intermediate bridge, the concentration of *p*-acetaminophenol and its interaction time with g-CNQDs also had a significant effect on the fluorescence intensity of the whole sensing system. Thus, the concentration of *p*-acetaminophenol and the reaction time of the advocated sensing system were optimized, as shown in Fig. S3.† It is easy to see that the fluorescence intensity of g-CNQDs was significantly enhanced when *p*-acetaminophenol was added to the g-CNQD fluorescence probe. The fluorescence intensity was proportional to the concentration of *p*-acetaminophenol. The fluorescence intensity reached the highest when the concentration was 1 mmol L^−1^. The corresponding fluorescence intensity will exceed the maximum value of the instrument when the concentration is further increased, therefore, 1 mmol L^−1^*p*-acetaminophenol was selected as the optimal concentration. At the same time, the stability of the interaction time of *p*-acetaminophenol with g-CNQDs on the fluorescence intensity was also studied, as shown in Fig. S3B.† The results indicated that the fluorescence intensity of g-CNQDs was unstable in the range of 0–2 min. The fluorescence intensity of g-CNQDs tended to remain stable with the further extension of reaction time between *p*-acetaminophenol and g-CNQDs. Therefore, the optimal reaction time was chosen as 3 min.

The reaction time between *p*-acetaminophenol and Cr^3+^ was studied, as shown in Fig. S4.† The results indicated that the response of g-CNQDs@*p*-acetaminophenol to Cr^3+^ was very sensitive; the fluorescence quenching could be completed within 1 min, and the fluorescence intensity has a strong stability for 10 min.

### 3.6 Selectivity of the Cr^3+^ fluorescence sensing system

**Fig. 6:**
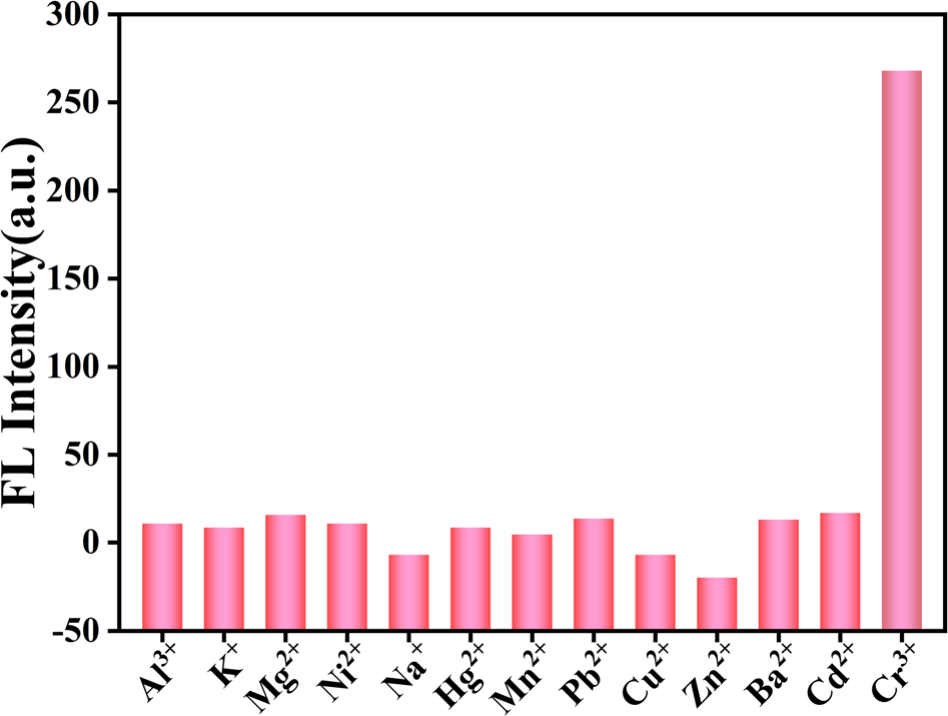
Fluorescence response of g-CNQDs in the presence of various mental ions.

Selectivity experiments play an important role in the sensing system. Based on the excellent optical properties of g-CNQDs, the selectivity of g-CNQDs@*p*-acetaminophenol fluorescence sensing system was implemented. The effect of different ions on fluorescence intensity was further explored, as shown in Fig. 6. Some interfering ions (Al^3+^, K^+^, Mg^2+^, Ni^2+^, Na^+^, Hg^2+^, Mn^2+^, Pb^2+^, Cu^2+^, Zn^2+^, Ba^2+^, and Cd^2+^) were used as control solutions. Then, the above solutions were added sequentially to the optimized mixed solution for fluorescence detection. The results showed that only Cr^3+^ showed significant fluorescence quenching on the g-CNQDs@*p*-acetaminophenol system in the presence of 100 times the Cr^3+^ concentration of interfering ions. This meant that the effect of other ions on the fluorescence intensity was almost negligible. These results indicated that the g-CNQDs@*p*-acetaminophenol system has excellent selectivity for Cr^3+^.

### 3.7 Construction of sensitivity and linear relation of the sensing system

**Fig. 7:**
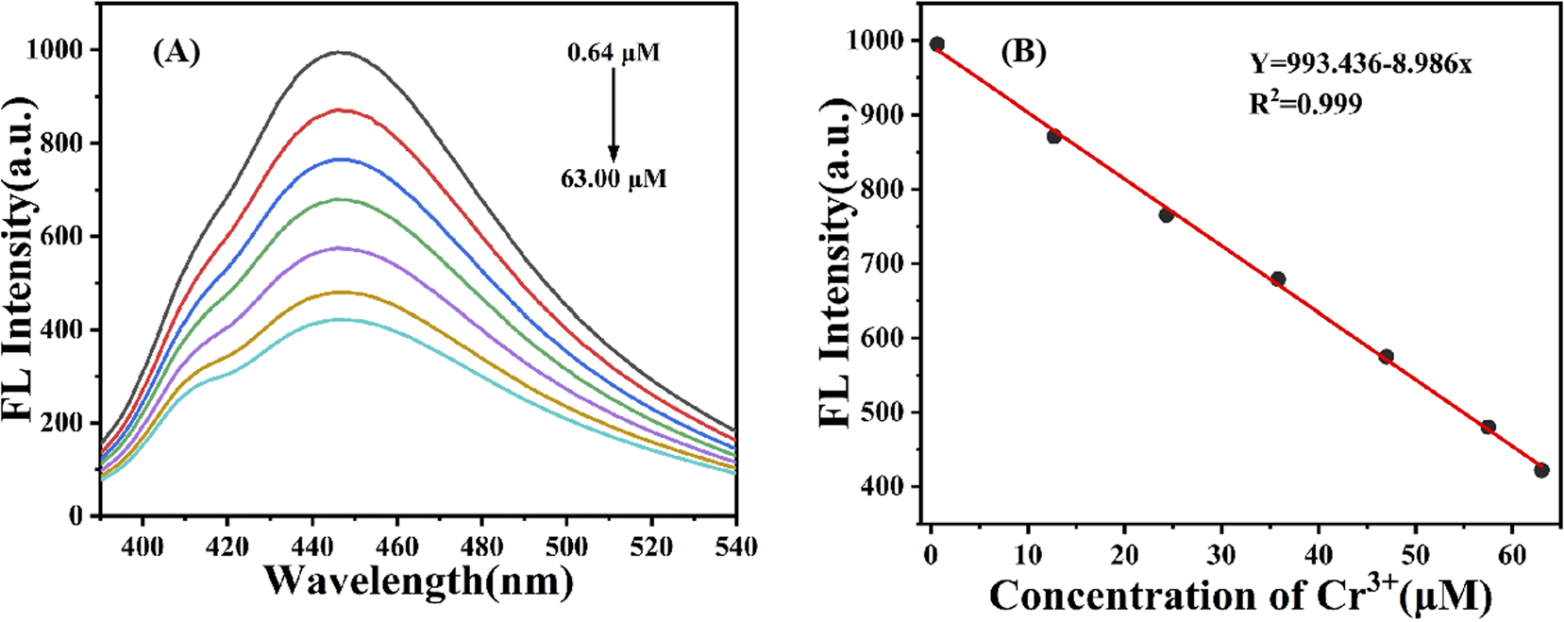
(A) Fluorescence spectrum of g-CNQDs@*p*-acetaminophenol with the addition of various concentrations of Cr^3+^ (0.64 μM to 63.0 μM); (B) linear relationship between fluorescence intensity and different concentrations of Cr^3+^.

To further investigate the sensitivity of the g-CNQDs@*p*-acetaminophenol fluorescence sensing system to Cr^3+^, the quenching effect of different concentrations of Cr^3+^ in the solution was evaluated. Fig. 7A shows the corresponding changes in the fluorescence intensity of the sensor system after the addition of different concentrations of Cr^3+^. The fluorescence intensity of the sensing system gradually decreased with the increase in Cr^3+^ concentration. The concentrations were 0.64, 12.71, 24.3, 35.8, 47.0, 57.5, and 63.0. This phenomenon indicated that g-CNQDs@*p*-acetaminophenol system was sensitive to Cr^3+^ concentration, which further confirmed the sensitivity of Cr^3+^ as an “off” fluorescent probe.

Furthermore, the linear relationship between the corresponding Cr^3+^ concentration in the range of 0.64–63.0 μM and the fluorescence intensity of the g-CNQDs@*p*-acetaminophenol system was studied, as shown in Fig. 7B. The linear equation of the standard curve by fitting was *Y* = 993.436 − 8.986*x* (*R*^2^ = 0.999) with the linear ranges of 0.64–63.0 μM, and the detection limit was calculated to be 0.23 μM by 3*δ*/*k* (*δ*: the blank standard deviation; *k*: the slope of the linear fitting curve equation).

### 3.8 Analysis performance comparison

In addition, to examine the sensing performance of the fluorescent sensor in this experiment, the detection limit and linear range of the present study system were compared with other methods reported in the literature for the determination of Cr^3+^. The results are shown in Table S1.† As can be seen from the chart, the fluorescence sensor designed in this study had a wide detection range and low detection limit compared with the other sensing systems. This means that the fluorescence sensing system designed in this study can be applied to the detection of Cr^3+^ in complex environments, and it has potential application prospects in the long run.

### 3.9 Exploration of the fluorescence detection mechanism

**Fig. 8:**
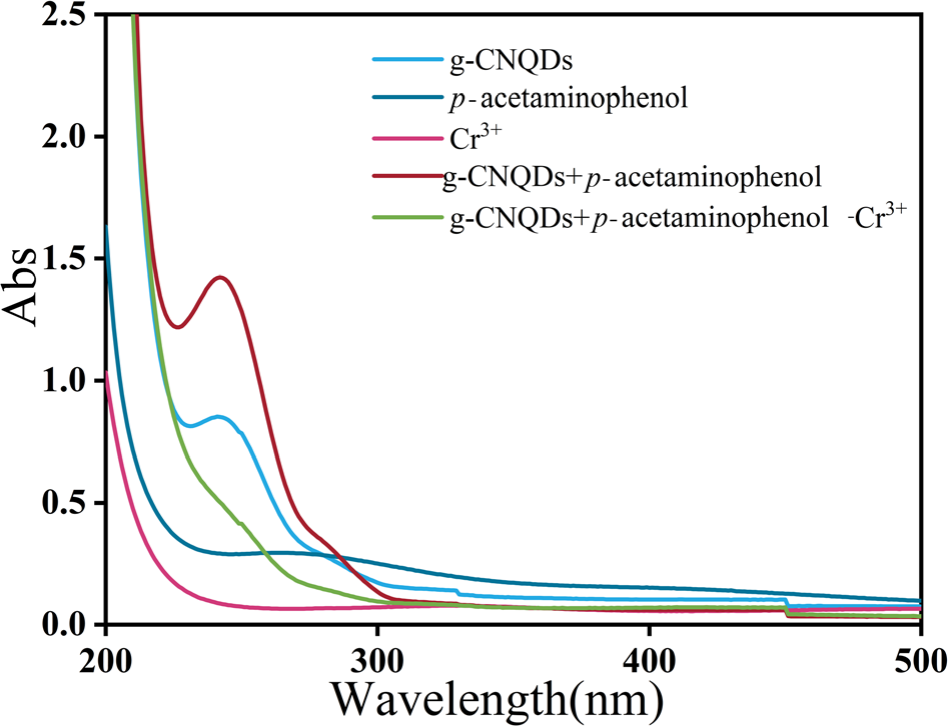
UV-visible absorption spectra of the multiple substances.

In order to verify the “on–off” mechanism in this sensing system, the UV-visible absorption spectra of g-CNQDs, *p*-acetaminophenol, Cr^3+^, g-CNQDs@*p*-acetaminophenol, and g-CNQDs@*p*-acetaminophenol–Cr^3+^ were studied. As shown in Fig. 8, neither *p*-acetaminophenol nor Cr^3+^ exhibited UV-visible absorption. Only g-CNQDs had a distinct UV-visible absorption peak at 254 nm. The UV-visible absorbance at 254 nm significantly increased when *p*-acetaminophenol was added to g-CNQDs. In the corresponding fluorescence spectrum, *p*-acetaminophenol also enhanced the fluorescence intensity of g-CNQDs. These phenomena suggest a possible synergistic effect between g-CNQDs and *p*-acetaminophenol. Therefore, the fluorescence signal of g-CNQDs is in the “on” state at this moment. The UV-visible absorbance peak at 254 nm was significantly reduced when Cr^3+^ was added to the g-CNQDs@*p*-acetaminophenol system. The corresponding fluorescence spectrum of g-CNQDs@*p*-acetaminophenol was also significantly reduced. These phenomena further prove that photoinduced electron transfer (PET) may have occurred with the addition of Cr^3+^. Therefore, the fluorescence signal of g-CNQDs is in the “off” state at this moment. Subsequently, the “on–off” fluorescence sensing system of g-CNQDs was constructed to realize the rapid detection of Cr^3+^.

### 3.10 Application of actual samples

Cr^3+^ is an essential trace element for the human body, which is a critical element for normal growth and development, and regulation of blood glucose. Therefore, it is necessary to evaluate the feasibility of the g-CNQDs@*p*-acetaminophenol fluorescence sensor for the detection of Cr^3+^ in actual samples. The accuracy and reproducibility of this analytical method were estimated by the standard recovery test. The fluorescence analysis of Cr^3+^ in tap water, bottled water, and river water was performed under optimized conditions. The experimental results are presented in Table 1. The recovery rates of Cr^3+^ were 97.40% to 103.87%. The above results indicated that the fluorescence quenching sensor for the quantitative detection of Cr^3+^ was accurate, reliable, and reproducible, and could be applied to the detection of Cr^3+^ in actual biological samples.

**Table 1:** Detection of Cr^3+^ in tap water

Samples	Added (μM)	Found^a^ (μM)	Recovery (%)	RSD^a^ (%)
Tap water	5	5.19	103.8	0.52
	15	14.70	98.0	0.30
	35	34.92	99.77	0.59
Bottled water	5	4.87	97.40	0.42
	15	15.11	100.73	0.39
	35	34.83	99.51	0.47
Serum	5	4.93	98.60	0.84
	15	15.06	103.87	0.40
	35	35.30	100.23	0.47

a Mean of three measurements.

## 4 Conclusions

In summary, g-CNQDs were synthesized using a modified low-temperature solid-phase reaction. The prepared g-CNQDs were of uniform size, good dispersion, and excellent water solubility. The g-CNQDs@*p*-acetaminophenol fluorescence sensing system showed remarkable selectivity for the detection of Cr^3+^. Further studies showed that g-CNQDs exhibited good salt tolerance and oxidation resistance in NaCl and H_2_O_2_ solutions. Meanwhile, based on the photoinduced electron transfer effect between Cr^3+^ and g-CNQDs@*p*-acetaminophenol can be used as a fluorescent sensor for the quantitative detection of Cr^3+^. The established fluorescence analysis method could be used for the detection of actual samples through the recovery of the added standards, indicating that this fluorescence sensing system was expected to be used for the real-time monitoring of Cr^3+^ in complex samples. Therefore, the sensor system has important practical significance for future applications in biological detection and environmental analysis.

## Author contributions

Xiaohua Xu: edited the manuscript, experimental scheme design, and formal analysis. Huye Li: conceptualization, experimental scheme design, reviewed the manuscript, and supervised the project. Yapeng Sun: formal analysis and edited the manuscript. Tianfeng Ma: experimental scheme design, performed the experiments and formal analysis. Lin Shi: experimental scheme design, performed the experiments and formal analysis. Wencheng Mu: experimental scheme design and formal analysis. Huan Wang: edited the manuscript and reviewed the manuscript. Yongchang Lu: reviewed the manuscript and supervised the project. All authors have read and agreed to the published version of the manuscript.

## Conflicts of interest

The authors declare no conflict of interest.

## Supplementary Material

RA-013-D3RA05091B-s001

## References

[cit1] Tian X.-M., Yao S.-L., Qiu C.-Q., Zheng T.-F., Chen Y.-Q., Huang H., Chen J.-L., Liu S.-J., Wen H.-R. (2020). Inorg. Chem..

[cit2] Cheng W., Tang P., He X., Xing X., Liu S., Zhang F., Lu X., Zhong L. (2021). Anal. Bioanal. Chem..

[cit3] Tsai M.-J., Liao K.-S., Hsu L.-J., Wu J.-Y. (2021). J. Solid State Chem..

[cit4] Yu Y. e., Wang Y., Yan H., Lu J., Liu H., Li Y., Wang S., Li D., Dou J., Yang L. (2020). Inorg. Chem..

[cit5] Paul S., Manna A., Goswami S. (2015). Dalton Trans..

[cit6] Krishnan U., Iyer S. K. (2022). J. Photochem. Photobiol., A.

[cit7] Fishbein L. (1987). Toxicol. Environ. Chem..

[cit8] Sharma A., Kapoor D., Wang J., Shahzad B., Kumar V., Bali A. S., Jasrotia S., Zheng B., Yuan H., Yan D. (2020). Plants.

[cit9] Rahman Z., Thomas L. (2021). Front. Microbiol..

[cit10] Kapoor R. T., Mfarrej M. F. B., Alam P., Rinklebe J., Ahmad P. (2022). Environ. Pollut..

[cit11] Roig-Navarro A., Martinez-Bravo Y., Lopez F., Hernandez F. (2001). J. Chromatogr. A.

[cit12] Arar E. J., Pfaff J. D. (1991). J. Chromatogr. A.

[cit13] Zayed M., Barsoum B., Hassan A. E. (1996). Microchem. J..

[cit14] Atikarnsakul U., Varanusupakul P., Alahmad W. (2018). Anal. Lett..

[cit15] Hagendorfer H., Goessler W. (2008). Talanta.

[cit16] Moreno F., Garcia-Barrera T., Gomez-Ariza J. (2010). Analyst.

[cit17] Zhang N., Suleiman J. S., He M., Hu B. (2008). Talanta.

[cit18] Chen H., Du P., Chen J., Hu S., Li S., Liu H. (2010). Talanta.

[cit19] Yilmaz E., Soylak M. (2016). Talanta.

[cit20] Sugiyama M., Fujino O., Kihara S., Matsui M. (1986). Anal. Chim. Acta.

[cit21] Ohira S.-I., Nakamura K., Chiba M., Dasgupta P. K., Toda K. (2017). Talanta.

[cit22] Tang X., Yu H., Bui B., Wang L., Xing C., Wang S., Chen M., Hu Z., Chen W. (2021). Bioact. Mater..

[cit23] Wang J., Zhou Y., Si G., Xu G., Zhou S., Xue X. (2023). J. Inorg. Biochem..

[cit24] Wang L., Xu D., Gao J., Chen X., Duo Y., Zhang H. (2020). Sci. China Mater..

[cit25] Fan Q., Dehankar A., Porter T. K., Winter J. O. (2021). Coatings.

[cit26] Xiang X., Tian L., Zhu X., Zhong Y., Xiao C., Chen L., Zhou S.-F. (2022). J. Electrochem. Soc..

[cit27] Deshmukh S., Pawar K., Koli V., Pachfule P. (2023). Acs Appl. Bio Mater..

[cit28] Huang Q., Bao Q., Wu C., Hu M., Chen Y., Wang L., Chen W. (2022). J. Pharm. Anal..

[cit29] Guo S., Zheng L., He W., Chai C., Chen X., Ma S., Wang N., Choi M. M., Bian W. (2023). Arabian J. Chem..

[cit30] Liu Z., Zhang X., Ge X., Hu L., Hu Y. (2019). Sens. Actuators, B.

[cit31] Cheng Q., Liu X., He Y., Ge Y., Zhou J., Song G. (2019). J. Fluoresc..

[cit32] Sun T., Yu X., Zhong S., Xu L., Zhao Y. (2020). J. Mater. Sci..

[cit33] Feng S., Pei F., Wu Y., Lv J., Hao Q., Yang T., Tong Z., Lei W. (2021). Spectrochim. Acta, Part A.

[cit34] Xie H., Fu Y., Zhang Q., Yan K., Yang R., Mao K., Chu P. K., Liu L., Wu X. (2019). Talanta.

[cit35] Ngo Y.-L. T., Chung J. S., Hur S. H. (2019). Dyes Pigm..

[cit36] Devi M., Das P., Boruah P. K., Deka M. J., Duarah R., Gogoi A., Neog D., Dutta H. S., Das M. R. (2021). J. Environ. Chem. Eng..

