# Supplementary material for: Novel “on–off” fluorescence sensing for rapid and accurate determination of Cr3+ based on g-CNQDs
Source: RSC Adv. 2023 Sep 28;13(41):28550–9. doi: 10.1039/d3ra05091b (PMC10534202; doi:10.1039/d3ra05091b)
Supplement: RA-013-D3RA05091B-s001 [file RA-013-D3RA05091B-s001.pdf]

Electronic Supplementary Material (ESI) for RSC Advances.
This journal is © The Royal Society of Chemistry 2023

Supplementary Material

A novel "on-off" fluorescence sensing for rapid and accurate determination of Cr^{3+} based on g-CNQDs

Xiaohua Xu[a], Huye Li[b], Yapeng Sun[c], Tianfeng Ma[a], Lin Shi[a], Wencheng Mu[a], Huan Wang[a,*] Yongchang Lu[a,*]

a. Modern Tibetan Medicine Creation Engineering Technology Research Center of Qinghai Province, China; College of Pharmacy, Qinghai Nationalities University, Xining 810007, China. E-mail: qhmuwh1028@126.com; qhlych@126.com.

b. The Fourth People's Hospital of Qinghai Province, Xining 810007, China.Address here.

c. *No. 2 Middle School in Xining City, Qinghai Province, Xining 810007, China.*

⁎ Corresponding authors.

E-mail addresses: qhmuwh1028@126.com; qhlych@126.com.

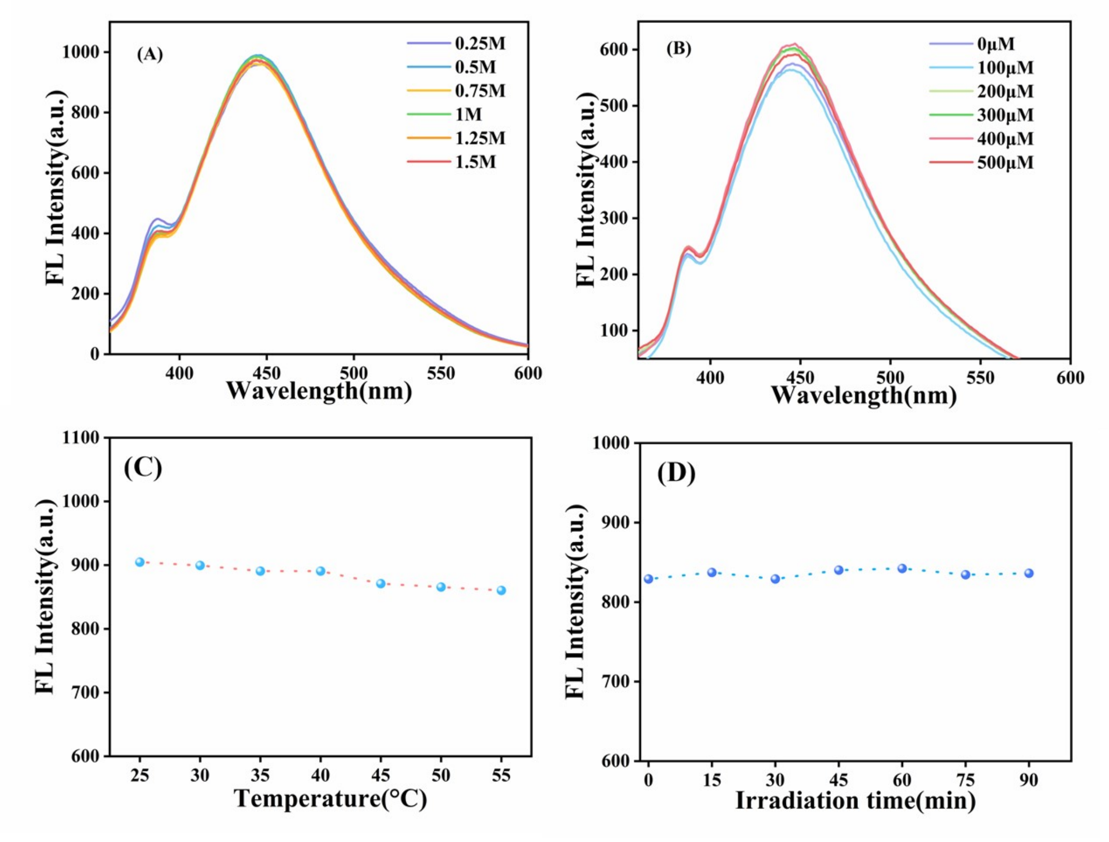

Fig. S1. (A) Effect of different concentrations of NaCl on the stability of g-CNQDs; (B) Effect of different H_2O_2 concentrations on the antioxidant capacity of g-CNQDs; Effect of (C) temperature and (D) duration of UV radiation on fluorescence intensity.

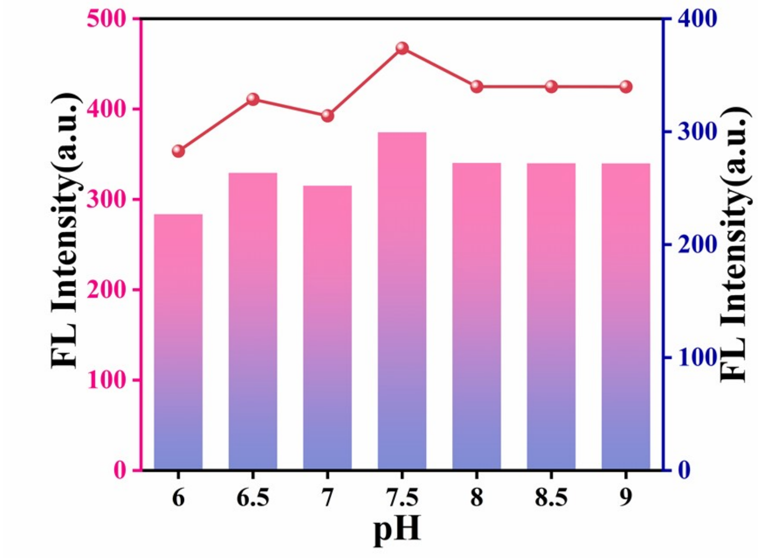

Fig. S2. Show the effect of different pH of PBS on the fluorescence intensity of g-

CNQDs system.

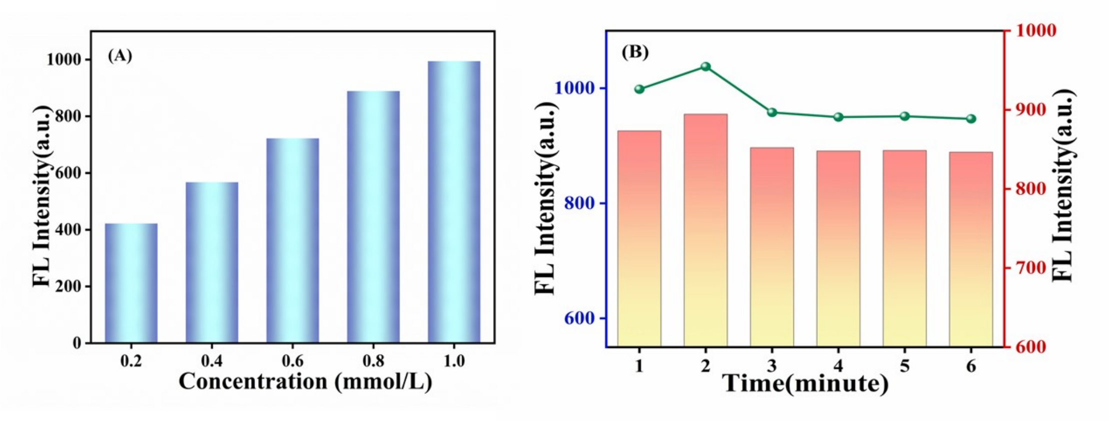

Fig. S3. (A) Effects of different concentrations of p-acetaminophenol and (B) reaction time of advocated system on fluorescence intensity.

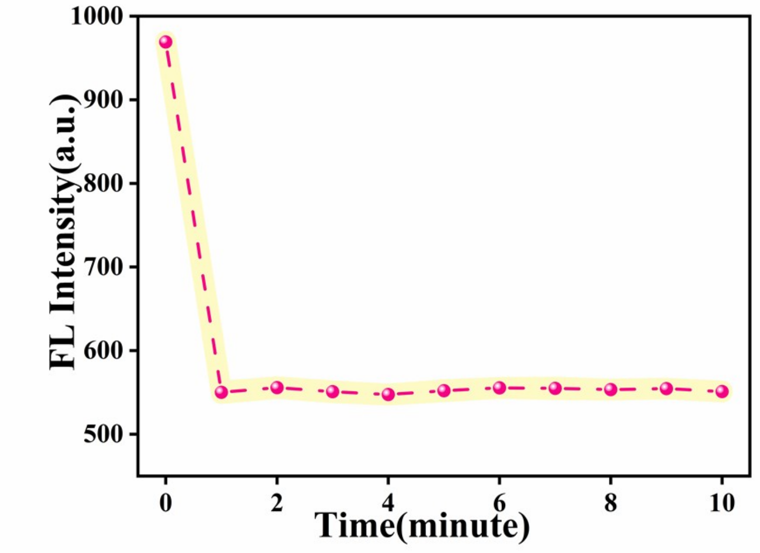

Fig. S4. Effect of incubation time with Cr^{3+} on fluorescence intensity of g-CNQDs@p-acetaminophenol.

Table S1. Comparison of detection of Cr^{3+} with various fluorescence probes

Fluorescent probe	Line range	LOD	Ref
AuNPs	1~11 μM	0.9 μM	[1]
Gold nanoparticles functionalized	20~25 μM	5 μM	[2]
2D podand fluorescent probes	_	17 μM	[3]
Pyridoxal conjugated AuNPs assay	7.5~1.3 × 10−5 M	11.5 μM	[4]
Rhodamine capped AuNPs assay	_	9.3 μM	[5]
Fluorescence sensing	0.64~63.0μM	0.23 μM	This work

References

[1] M. Cao, X. Ye, Y. Liu, P. Zhang and Z. Zhang, *Chem. Pap.*, 2023, 1-7.

[2] Z. Zhang, X. Ye, Q. Liu, Y. Liu and R. Liu, *J. Anal. Sci. Technol.*, 2020, **11**, 1-7.

[3] S. Panda, P. B. Pati and S. S. Zade, *Chem. Commun.*, 2011, **47**, 4174-4176.

[4] S. Bothra, R. Kumar and S. K. Sahoo, *New J. Chem.*, 2017, **41**, 7339-7346.

[5] N. Manjubaashini, T. D. Thangadurai, G. Bharathi and D. Nataraj, *J. Lumin.*, 2018, **202**, 282-288.
